# EphB3 signaling induces cortical endothelial cell death and disrupts the blood–brain barrier after traumatic brain injury

**DOI:** 10.1038/s41419-017-0016-5

**Published:** 2018-01-08

**Authors:** Poincyane Assis-Nascimento, Yanina Tsenkina, Daniel J. Liebl

**Affiliations:** The Miami Project to Cure Paralysis, Department of Neurological surgery, University of Miami Miller School of Medicine, 1095 NW 14th Terrace, R-48, Miami, FL 33136 USA

## Abstract

Damage to the cerebrovascular network is a major contributor to dysfunction in patients suffering from traumatic brain injury (TBI). Vessels are composed of lumen-forming endothelial cells that associate closely with both glial and neuronal units to establish a functional blood–brain barrier (BBB). Under normal physiological conditions, these vascular units play important roles in central nervous system (CNS) homeostasis by delivering oxygen and nutrients while filtering out molecules and cells that could be harmful; however, after TBI this system is disrupted. Here, we describe a novel role for a class of receptors, called dependence receptors, in regulating vessel stability and BBB integrity after CCI injury in mice. Specifically, we identified that EphB3 receptors function as a pro-apoptotic dependence receptor in endothelial cells (ECs) that contributes to increased BBB damage after CCI injury. In the absence of EphB3, we observed increased endothelial cell survival, reduced BBB permeability and enhanced interactions of astrocyte-EC membranes. Interestingly, the brain’s response to CCI injury is to reduce EphB3 levels and its ligand ephrinB3; however, the degree and timing of those reductions limit the protective response of the CNS. We conclude that EphB3 is a negative regulator of cell survival and BBB integrity that undermine tissue repair, and represents a protective therapeutic target for TBI patients.

## Introduction

Traumatic brain injury (TBI) is a devastating disorder that occurs when an external mechanical force causes injury to the brain, leading to dysfunction that can initially result from tearing of tissue, hemorrhage, and other physical damage^1^. TBI ranges from mild to severe and consists of a broad spectrum of symptoms and disabilities depending on the severity of the trauma. Inherent to most brain injuries is the disruption of blood vessels and the blood–brain barrier (BBB), which leads to brain edema and hematomas but also to secondary injury pathologies and overall neurological dysfunction^2–5^. The BBB is composed of high-density endothelial cells (ECs) that form tight junctions, a thick basal membrane as well as astrocytic and pericytic endfeet. There are several underlying events involved in cerebrovascular BBB alterations, including disruption of tight junction seals, widening of intercellular spaces, changes in endothelial transport properties, extracellular matrix degradation, dissociation of gliovascular cells, and peripheral cell infiltration^2,5,6^. Loss of brain ECs and pericytes can also contribute to vessel leakiness and breakdown of the BBB, which is accompanied by both extravasations of larger or hydrophilic circulating proteins as well as hypoxia^7^.

Erythropoietin-producing human hepatocellular (Eph) receptors make up the largest subfamily of tyrosine kinases receptors. Both Eph receptors and their ligands, ephrins, are membrane bound proteins that interact to initiate bidirectional signals in both the ligand- and receptor-containing cells^8,9^. The family consists of two subclasses, namely A and B class, mainly separated by ligand structural differences and binding preferences. Ephrins and Eph receptors are expressed in nearly all tissues of the mammalian embryo, and participate in a wide spectrum of developmental, homeostatic, and pathological processes^10^. In the arterial-venous system, blood vessels express several ephrins and Eph receptors to regulate a variety of critical processes, including angiogenic remodeling, pathological vasculogenesis, angiogenesis, and neovascularization^11^. In particular, ephrinB2 and EphB4 are essential for arterial-venous specification and vascular remodeling^12–14^. In fact, germ-line deletion of ephrinB2 results in embryonic lethality as a result of underdeveloped vessels and poor vascular organization^11^. More recently, ephrinB2 has been shown to participate in vessel wall assembly and establishment of proper EC-pericyte interactions^15^, and EphB4 in the angiogenic responses of endothelial progenitor cells (EPCs)^16^. Other Eph receptors have also been shown to regulate vascular angiogenesis in the developing nervous system, namely EphB2, EphB3, and EphA4 receptors^11,17,18^.

In recent years, Eph receptors have been found to have pro-apoptotic responses following traumatic central nervous system (CNS) injury^19–21^, and are now classified as new members of a larger “dependence receptor” family^22^. Dependence receptors are transmembrane proteins that have dual opposing roles depending on the availability of their corresponding ligand. In the absence of their ligand(s) occurring under stressful conditions, dependence receptors induce apoptotic cell death characterized by proteolytic cleavage of Eph receptors leading to changes in its protein conformation and the release/exposure of an addiction/dependence domain^23^. When the ligand is present, these receptors can promote normal development and tissue homeostasis by inducing ligand-mediated positive signals^24,25^. Currently, two Eph receptors, EphA4 and EphB3, have been found to have dependence receptor functions in the naive and injured adult CNS^20,21,23,26^. Here, we describe a new dependence receptor role for EphB3 in regulating cerebral vascular EC survival after TBI. We also demonstrate that ephrin–EphB3 interactions regulate BBB stability after TBI.

## Material and methods

### Animals

Adult C57BL/6 male mice ages 2–4 months were used for all experiments. Cdh5^-^zG mice were generated by crossing Cdh5 (pac)-CreERT2 (Tg (Cdh5^-^cre/ERT2) 1Rha, MGI: 3848982)^27^ with Rosa zGreen reporter mice (007906 B6.Cg-Gt (ROSA) 26Sor<tm6 (CAG-ZsGreen1) Hze>/J; The Jackson Laboratory, Bar Harbor, ME). Thy-1-YFP mice were purchased from Jackson Laboratory (JAX Mice Database-003782 B6.Cg-Tg (Thy-1-YFP) HJrs/J). The generation of ephrinB3 knockout (ephrinB3^-/-^) and EphB3 knockout (EphB3^-/-^) mice and genotyping using PCR analysis has been previously described^28–30^. Cdh5^-^zG-ephrinB3^-/-^ and Cdh5-zG-EphB3^-/-^ mice were generated by crossing the Cdh5-zG mice with the ephrinB3^-/-^ and EphB3^-/-^ mice. Animals were housed in a 12 h light/dark cycle and food and water were supplied ad libitum. All procedures related to animal use and care were approved by the University of Miami Animal Use and Care Committee.

### Surgeries

In preparation for CCI injury, mice were anesthetized with 100 mg/kg ketamine and 10 mg/kg xylazine by intraperitoneal (i.p.) injections. A 5 mm craniotomy was aseptically made using a portable drill over the right parieto-temporal cortex (−2.5 mm caudal and 3 mm lateral from bregma, epicenter). The injury was generated using a 3 mm beveled stainless steel tip piston attached to an eCCI-6.3 device (Custom Design & Fabrication, Panama City, FL, USA), at 4 m/s velocity, depth of 0.5 mm and impact duration of 15 ms. Surgical sham mice received only the opening and re-suturing of the skin. After CCI injury the skin was sutured using 5–0 coated vicryl sutures (Ethicon, Mokena, IL, USA) and animals were placed on a warm heating pad until fully recovered from anesthesia. Buprenorphine (0.1 mg/kg) and saline were administrated to animals post-surgery. For cell death rescue analysis, Alzet mini osmotic pumps (Alzet Durect Corp, Cupertino, CA, USA) were preloaded with recombinant ephrinB3 proteins (100 μg/mL) or phosphate buffer saline (PBS) vehicle, placed directly over the injury using a stereotactic holder, and secured to cranium with surgical glue (Locite 454 Prism Surf 3G, Rocky Hill, CT, USA). Pumps were placed under the skin of the dorsal neck region for an infusion over a 24-hour period (8 μL/hr rate; 80 μg/kg/day ephrinB3).

### Tamoxifen treatment

Adult Cdh5-zG male mice received six i.p. injections of 50 mg/mL Tamoxifen (Sigma, St. Louis, MO, USA) diluted in 10% absolute ethanol/90% sunflower oil (Sigma). The treatments were administered daily over an 8-day period, with the exception of days 2 and 3, starting 15−days prior to experimentation. Animals were used experimentally 1 week after the last injection.

### Primary mouse ECs and human umbilical vein endothelial cell (HUVEC) cultures

The protocol for culturing primary cortical ECs was adapted from previously described methods^31,32^. The brains from six adult wild-type (WT) mice were extracted and placed in cold Minimum Essential Medium (MEM-HEPES, Sigma), following euthanization using ketamine/xylazine cocktail. Meninges, cerebellum, olfactory bulbs, and midbrain were removed and the cortices were dissected, minced into small pieces, and then incubated with 30 U/mL papain (Worthington, Lakewood, NJ, USA) and 40 μg/mL DNase I (Worthington) in Earl’s Balanced Salt Solution (EBSS, Worthington) for 70 min at 37 °C. Following incubation the digested brain tissue was passed ten times through an 18-gauge needle (Becton Dickinson (BD), Franklin Lakes, NJ, USA) and successively ten times through a 21-gauge needle (BD) until fully homogenized. The dissociated tissues were then mixed with 1.7 volumes of freshly prepared, ice cold 22% bovine serum albumin (BSA in PBS pH 7.4, Sigma) and centrifuged at 2600 rpm for 10 min at 4 °C. After centrifugation a thick myelin/lipid layer formed on the top of the vial, which was carefully aspirated and discarded. The blood vessel pellet was washed in 5 mL of freshly prepared endothelial cell growth medium (ECGM) consisting of 40 µg/mL heparin (Sigma), 2.5 µg/mL l-ascorbic acid (Sigma), 4 mM L-glutamine (Sigma), 37.5 µg/mL endothelial cell growth supplement (Millipore, Billerica, MA, USA), 1% penicillin/streptomycin (Sigma), and 10% fetal bovine serum, (Hyclone, South Logan, Utah, USA) all diluted in Ham’s F12 media (Sigma). Cells were resuspended in 4 mL ECGM and platted onto two wells (2 mL per well) of a 6-well plate coated with rat tail collagen type I (Sigma) and incubated at 37 °C at 5% CO_2_. Twenty-four hours post seeding, cells were washed once with pre-warmed Ham’s F12 and media was replaced with fresh ECGM containing 4 μg/mL puromycin (Axxora, Farmingdale, NY, USA) and incubated for 3 days. Puromycin is an inhibitor of protein synthesis inducing cell death; however, cerebrovascular endothelial cells (cvECs) are protected because they express high levels of the multi-drug (MDR) resistance proteins. This lead to selective killing of non-cvECs. After 3 days cells were washed once again with pre-warmed Ham’s F12 and media to remove the puromycin and replaced with fresh ECGM. Cells were allowed to reach confluency and passaged twice prior to being used for quantitative PCR analysis.

HUVECs were a kind gift from Dr. Claudia Rodrigues (University of Miami, Miami, USA). The cells were cultured using endothelial growth media bullet kit (Lonza, Allendale, NJ, USA) following the manufacturer’s instructions on collagen type I-coated plates (Corning® Biocoat^TM^, Tweksbury, MA, USA). EphB3 protein expression was detected using a standard western blot procedure on HUVECS lysates using a primary mouse anti-EphB3 antibody (diluted 1:200 Novus Abnova, Littleton, CO, USA). The membrane was incubated at 4 °C overnight following by incubation with a HRP-conjugated secondary anti-mouse antibody (diluted 1:5000 Jackson ImmunoResearch Inc. Laboratories, West Grove, PA, USA) for 2 h at room temperature. The membrane was developed using SuperSignal West Pico Chemiluminescent Substrate (Pierce Biotechnology, Rockford, IL, USA). To evaluate whether ephrinB3 promotes HUVEC survival under stressful conditions, growth factors were removed (GF-) from the culture media over a 48-hour period. GF(-) cultures received 1 µg/mL ephrinB3 or vehicle every 12 h, and cell survival was assessed at 48 h using trypan blue (Sigma) cell exclusion assay and an automatic cell counter (Bio-Rad, Hercules, CA, USA).

### Flow cytometry

At either 1 or 3 days post-injury (dpi), mice were euthanized using ketamine/xylazine cocktail overdose. Brains were immediately extracted and placed in cold Hank’s Balanced Salt Solution, without calcium chloride, magnesium chloride or magnesium sulfate (HBSS^−/−/−^,Gibco, Langley, OK, USA). The injured cortex or the corresponding sham cortices were dissected and digested in 30 U/mL papain (Worthington) and 40 μg/mL DNase I (Worthington) in EBBS (Worthington) for 70 min at 37 °C. The brain tissue was dissociated ten times through 18-gauge and 21-gauge needles (BD) until fully homogenized. For myelin removal, brain cells were mixed with 1.7 volumes of 22% BSA in PBS pH 7.4 and centrifuged at 2600 rpm for 10 min at 4 °C. Cortical cell homogenates were re-suspended in 1 mL HBSS^+/+^ (Gibco) solution and stained with live/dead fixable Near-IR dead Cell Stain (Life Technologies, Eugene, OR, USA) for 30 min on ice. Cells were blocked in FcR solution (MACS Miltenyi Biotec, Auburn, CA, USA in 0.5% BSA) for 15 min at 4 °C and pre-conjugated antibodies were then subsequently added for surface staining for 20 min at 4 °C. The primary antibodies included: 1:100 PE-Cy7 anti-mouse CD45 (ThermoFischer, Rockford, IL, USA), 1:200 PE anti-mouse CD133 (Biolegend, San Diego, CA, USA), and 1:100 BV421 rat anti-mouse CD144 (VE-Cadherin) (BD Horizon, San Jose, CA, USA), all diluted in FcR blocking solution. For intracellular staining, cells were fixed for 20 min with Cytofix on ice (BD Horizon) and incubated with anti-mouse vascular endothelial growth factor receptor-2 (VEGFR-2) antibody (CD309–Cell Signaling, Danvers, MA, USA) diluted 1:300 in BD perm/wash buffer for 20 min at RT. Cells were then incubated with the secondary antibody donkey anti-rabbit Alexa Fluor 594 IgG (ThermoFischer) diluted 1:500 in BD perm/wash buffer for 30 min at room temperature. Cells were resuspended in 0.5 mL flow cytometry staining buffer (ThermoFischer). Approximately 10 to 15 min prior to analysis, the samples were transferred to BD TruCount tubes (BD Biosciences, San Jose, CA, USA) and run on a special order BD LSRII flow cytometer configured with a 405, 488, 532, and 640 nm laserline using BD FACS Diva 8.0.1 software. Data were analyzed in Kaluza 1.3 (Beckman Coulter, Brea, CA, USA). Fluorescence minus one staining and the corresponding isotype controls were used to determine positive staining from background for all antibodies.

For infiltration studies *s*ham and CCI injured mice were processed as described above. Briefly, after the L/D stain FcR blocking steps, the cells were incubated for 20 min at 4 °C with 1:100 PE-Cy7 anti-mouse CD45 (ThermoFischer) and 1:200 BV-650 anti-mouse CD11b (Biolegend) pre-conjugated antibodies for surface staining diluted in FcR blocking solution and protected from light. Approximately 10 to 15 min prior to analysis, the samples were transferred to BD TruCount tubes (BD Biosciences) to be analyzed by flow cytometry.

### Fluorescence-activated cell sorting (FACS)

Sham and CCI injured tissues were prepared as for flow cytometry at 1 dpi as described above. Cortical cells were incubated for surface staining with PE-Cy7 anti-mouse CD45 (ThermoFischer) 1:100 and BV421 rat anti-mouse CD144 (VE-Cadherin) (BD Horizon) 1:100 pre-conjugated antibodies, for 20 min at 4 °C, diluted in FcR blocking solution. Cells were resuspended in 0.5 mL flow cytometry staining buffer (ThermoFischer) and run on a Beckman Coulter MoFlo Astrios EQ using a 100 μm nozzle at 25 psi at a sort rate of about 10,000 events/second using IsoFlow (Beckman Coulter). Debris were gated out using a Forward Scatter Area x Side Scatter Area plot. Aggregates were excluded using a Forward Scatter Height x Forward Scatter Width and a Sideward Scatter Height x Sideward Scatter Width plot. CD45^+^ cells were excluded and cvECs were sorted based on BV421 expression using CD45 PE-Cy7 log Area by a CD144 BV421 log Area plot. Post sort purities for CD45^-^/CD144^+^ cvEC population was >95%. Cells were collected directly into 250 μL TRI Reagent (Zymo Research, Irvine, CA, USA) for subsequent RNA extraction.

### RNA extraction and quantitative reverse transcriptase PCR (qRT-PCR) analysis

Sorted cvECs were processed for RNA extraction and purification using Direct-zol RNA Mini-Prep kit (Zymo Research) and reverse transcriptase (RT) reactions Omniscript RT kit (Quiagen, Hilden, Germany) were performed according to manufacturer’s instructions. No RT samples were used as negative control for each animal. Samples were then prepared for qPCR analysis using the Maxima SYBR Green qPCR kit (Thermo Scientific, Wilmington, DE, USA) on 96-well plates (Bio-Rad) and covered with adhesive films (VWR, Radnor, PA, USA). Samples were run on an Eppendorf Mastercycler EP Realplex (Quiagen) and analyzed using Realplex software version 2.2. Delta (∆) Ct was calculated by subtracting the corresponding GAPDH Ct from each sample Ct and data were presented as 2*^-∆Ct^ expression. The qPCR primers used are listed on Table 1. All primers were designed using Primer3 software^33^ integrated into the Primer-BLAST web service (http://www.ncbi.nlm.nih.gov/tools/primer-blast)^34^. The primers were designed to span over exon–exon junctions in order to avoid amplification of contaminant genomic DNA and pre-mRNA. In order to ensure generation of a single amplicon per qPCR reaction, the primers were selected based on the melting curve analysis performed using Realplex software version 2.2 (Quiagen).

**Table 1:** Primer sets for qPCR analysis

Primer name	Size	Sequence
*ephrinB3*	112 bp	5′: GGGCCAGGGGGTGTG
		3′: GCCTGGAACCTCTTATTCGC
*EphB3*	160 bp	5′: CTCCACTGTAACCAGCCAG
		3′: TGGGCACCTGAACCTCTTTC
*GAPDH*	92 bp	5′: GAGGCCGGTGCTGAGTATGTCGTG
		3′: TCGGCAGAAGGGGCGGAGATGA

### Cell proliferation

Cell proliferation was assessed using the Click-it EdU labeling kit (Life Technologies) in Alexa Fluor (AF)-647 for flow cytometry. Mice were pulsed with 3 i.p. injections of 50 mg/kg EdU (Life Technologies) on days 1, 2 and 3 following CCI or sham surgery and tissue was processed at 3 dpi. EdU staining was performed according to the manufacturer’s instructions after the CD309 intracellular staining step on fixed and permeabilized cells and then transferred to BD TruCount tubes (BD Biosciences) to be analyzed by flow cytometry as described above.

### Deoxynucleotidyl transferase-dUTP nick end labeling (TUNEL) IHC

WT, ephrinB3^-/-^ and EphB3^-/-^ sham or CCI injured animals were anesthetized at 1 dpi and received intracardiac perfusion with PBS and 4% PFA. Thirty micron stereological cryostat sectioned brain tissues were washed with PBS for 10 min at room temperature and then permeabilized with 1% Triton-X in PBS for 30 min, blocked with 5% BSA in PBS for 30 min at room temperature, and immunostained with GLUT-1 (Glucose Transporter-1) rabbit Polyclonal (Millipore) antibody overnight at 4 °C, diluted 1:100 in 5% BSA in PBS pH 7.4. To ensure proper antibody cross-linking to the tissue, sections were post-fixed in 4% PFA for 15 min at room temperature, then permeabilized for 5 min at −20 °C with a 2:1 ratio ethanol:acetic acid solution. Following 2X PBS washes, sections were pre-treated with Proteinase K buffer (1 M Tris pH 8.0 and 0.5 M EDTA pH 8.0) for 10 min at room temperature, then incubated with 12 mg/mL Proteinase K enzyme diluted in Proteinase K buffer (20 μl/mL) for 15 min. Sections were washed with 2X PBS for 5 min/each, equilibrium buffer (Apoptag Red In Situ Apoptosis detection kit, Millipore) was added for 15 min at 37 °C in humidified chamber, then TdT enzyme diluted in reaction buffer was added for 1 h at 37 °C in a humidified chamber. Stop/Wash Buffer was added to all sections for 10 min at room temperature followed by 3X PBS washes for 1 min each. Working strength A594 anti-digoxigenin conjugate, combined with 1:500 Donkey anti-Rabbit A488 (Life Technologies) secondary antibody was applied to each section for 30 min at room temperature in a humidified chamber. Sections were washed 3X PBS and 1:500 Hoechst nuclear stain (Sigma) diluted in dH_2_O for 10 min at room temperature and mounted with Fluorogel (Electron Microscopy Sciences, Hatfield, PA, USA). For the cell death rescue analysis, WT and EphB3^-/-^ CCI injured animals were infused with either vehicle (PBS) or recombinant ephrinB3 protein for 24 h and processed as described above.

### Stereological assessment of cell death

Unbiased stereological analysis of Glut-1^+^/TUNEL^+^ cvECs at the injury penumbra was assessed using MicroBrightField StereoInvestigator software package (MBF Bioscience, Williston, VT, USA) and an Olympus BX51 microscope (Olympus America, Center Valley, PA, USA) equipped with a CCD camera at 63X objective. Four 30 μm sections, 250 μm apart encompassing levels −1.6 mm to −2.6 mm from bregma, were quantified per animal using Stereoinvestigator. The ipsilateral cortical region stretching from the innermost corner of the dentate gyrus to the outermost boundary of CA3 was contoured at 4X magnification. At 63X (NA1.42) a sampling grid of 75 × 75 μm was placed over the region of interest and Glut-1^+^/TUNEL^+^ cells were counted within a 25 × 25 μm frame. Data presented as estimated counts per 100 μm^3^ and normalized to WT sham controls.

### Evans blue extravasation assay

A 0.5% sterile Evans blue (Sigma) solution was prepared in PBS and passed through a 0.2 μm filter to remove any particulate matter that has not dissolved. Mice having undergone either sham or CCI surgery were injected with 200 μL Evans blue either intravenously (i.v.) or i.p. No differences were observed between using i.v. or i.p. administration of Evans blue (not shown). Anesthetized sham or CCI injured animals received intracardiac perfusion with PBS, pH 7.4, 3 h after the Evans blue injection to remove any excess dye. Brains were removed and the ipsilateral and contralateral hemispheres were incubated separately in 500 μL Formamide (Thermo Fischer) for 24 h at 55 °C. Samples were centrifuged to pellet the tissue and the supernatant absorbance was measured at 610 nm using a NanoDrop 1000 Spectrophotometer (Thermo Scientific). Formamide was used to blank the instrument. Evans blue absorbance was normalized to the contralateral hemisphere for each animal.

### Vessel area and volume fraction

Cdh5-zG-WT, Cdh5-zG-ephrinB3^-/-^, and Cdh5-zG-EphB3^-/-^ mice were treated with tamoxifen as described above. Sham or CCI injured animals were anesthetized and received intracardiac perfusion with PBS and 4% PFA. Brains were removed, post-fixed in 4% PFA at 4 °C overnight, incubated for 24 h in a 30% sucrose solution at 4 °C, then embedded in 30% sucrose clear frozen section compound (VWR) for cryostat sectioning and kept at −80 °C. Thirty-micron stereological cryostat sections were washed 3X with PBS for 5 min each wash and mounted with Fluorogel (Electron Microscopy Sciences). Six non-overlapping confocal images, taken at 60X magnification, of the vessels in the penumbra region were randomly obtained for each CCI injured animal or corresponding region on sham controls. Images were acquired on an Olympus FV1000 confocal microscope with 5-channel detection and spectral unmixing modes equipped with 458, 488, 514, 543, and 635 nm laser lines. Vessel area fraction was computed using the surface-tracing feature of Imaris 8.1.2 3D image analysis software (Bitplane, Concord, MA, USA). Automatic surface segmentation was conducted on Cdh5-zG positive expression and set with surface area detail of 0.414 μm. Segmentation was done with specific threshold levels kept constant across all images and was originally determined according to control. Vessel area fraction was determined as the rendered surface area per 100 μm^2^.

### Analysis of membrane interactions

Sham or CCI injured animals were anesthetized, perfused, processed for cryostat sectioning, and immunostained as described previously. 1:200 rabbit monoclonal anti-PDGFRβ antibody clone Y92 (Abcam, Cambridge, MA, USA) was used to label pericytes and 1:500 rabbit polyclonal anti-GFAP antibody was used to label astrocytes (DAKO, Santa Clara, CA, USA) and reacted with fluorescently-labeled goat anti-rabbit A647 F(ab’) 2 Fragment (Life Technologies) secondary antibody diluted 1:500 in 5% BSA. Six non-overlapping confocal images were obtained of the penumbra region for each CCI injured animal or corresponding region on sham controls. Images were acquired on an Olympus FV1000 confocal microscope (Olympus America) with five channel detection and spectral unmixing modes equipped with 458, 488, 514, 543, and 635 nm laser lines. Coloc2 plugin of FIJI-ImageJ imaging analysis software^35^ was used to quantify the amount of overlap between the two channels. Coloc2 uses pixel intensity spatial correlation for analysis, automatic thresholding, and significance testing. The Mander’s split colocalization coefficients determine the proportion of signal in a channel, which colocalizes with the other channel. Z-stack images were used for analysis. All images were acquired using identical parameters including the spatial sampling rate, laser intensities, PMT, and offset levels to avoid different signal:noise levels. Coloc2 parameters were set as PSF = 3 and run iterations set as 10 for robust Costes auto threshold determination. This method determines which threshold pair gives a Pearson’s correlation coefficient of zero for the pixels below the thresholds and is fully reproducible among similar data sets. Results were graphed as Mander’s Colocalization coefficients and normalized to WT sham control for each group.

### Statistical analysis

Unpaired two-tailed Student’s *t*-test with 95% confidence interval was used to compare cell populations from sham and CCI injured animals for the studies where only those two groups were being compared. One-way ANOVA with either Bonferroni’s or Newman-Keuls multiple comparison post-hoc tests were used for statistical analyses including three or more experimental groups. Statistical analyses were performed with GraphPad Prism software (GraphPad Software Inc., San Diego, CA, USA), version 5.0 where error bars represent ± 1 standard error of the mean (SEM) for all graphs. *P* < 0.05 were considered significant for all comparisons.

## Results

### Improved cortical vascular endothelial cell (cvEC) numbers and vessel density in the absence of EphB3 after CCI injury

**Fig. 1:**
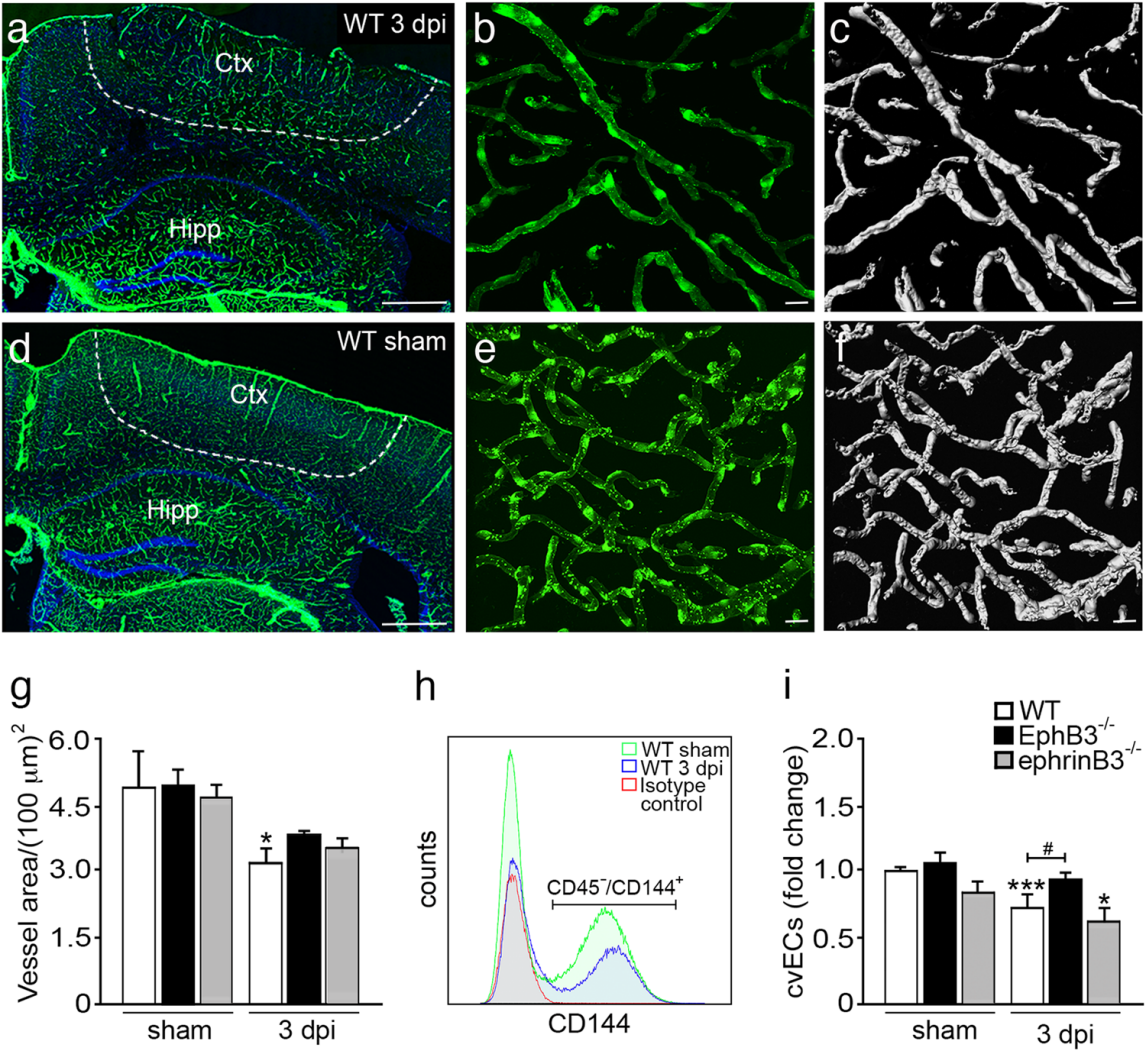
CCI injury led to reduced vessel density and cortical vascular endothelial cells (cvECs) in the absence of EphB3 **a** Low-magnification representative image of a Cdh5-zG WT cortex at 3 dpi, where dash line outlines the injury penumbra. High-magnification representative image of Cdh5-zG expression in cvECs **b** and 3D Imaris reconstructed image **c** for vessel area measurements in the injury penumbra. **d** Low-magnification representative image of a sham Cdh5-zG WT cortex, and high-magnification representative image of Cdh5-zG expression in cvECs **e** and 3D Imaris reconstructed image **f**. **g** Measurements of vessel area showed a significant reduction in CCI injured WT mice (*P* < 0.05) as compared to sham controls. *N*-values for panel **g** are as follows: WT sham (*n* = 10); WT CCI (*n* = 12); EphB3^-/-^ sham (*n* = 10); EphB3^-/-^ CCI (*n* = 13); ephrinB3^-/-^ sham (*n* = 7); ephrinB3^-/-^ CCI (*n* = 9). **h** Flow cytometry overlay histogram (gated on CD45^-^ cells) for the analysis of CD45^-^/CD144^+^ cvECs from WT mice showed separation from CD144^-^ cells and difference between sham (green), CCI injured (blue), and isotype control (red). **i** Flow cytometry counts showed reduced numbers of cvECs in WT and ephrinB3^-/-^ cortices, but not the EphB3^-/-^ cortex. *N*-values for panel **i** are as follows: WT sham (*n* = 12); WT CCI (*n* = 15); EphB3^-/-^ sham (*n* = 5); EphB3^-/-^ CCI (*n* = 6); ephrinB3^-/-^ sham (*n* = 14); ephrinB3^-/-^ CCI (*n* = 15). *^,#^*P* < 0.05; ***P < 0.001. *Compared to their respective genotype specific controls. ^#^Compared to WT CCI injured mice. Bar is 500 μm in **a**, **d** and 20 μm in **b, c, e, f**

To evaluate whether EphB3 regulates cortical vessel integrity after CCI injury, we examined vessel density in sham cadherin5-zGreen (cdh5-zG) reporter mice at 3 days post-CCI injury (dpi) (Fig. 1). Cadherin-5 or vascular endothelial (VE)-cadherin is expressed in all viable ECs where green fluorescence is observed following tamoxifen administration. We performed non-biased stereological measurements of vessel area in moderate CCI and sham injured WT, EphB3^-/-^, and ephrinB3^-/-^ mice (Fig. 1). Low-magnification images of the WT injured cortical penumbra (Fig. 1a; dash line) shows reduced vessel density at 3 dpi as compared to a similar region of the WT sham cortex (Fig. 1d). High-magnification images of the vascular network show vessels made up of ECs that form a vessel lumen (Fig. 1b, e), where the surface-tracing feature of Imaris 3D analysis was used to compute vessel area (Fig. 1c, f). CCI injury leads to reduced vessel density (Fig. 1b, c) as demonstrated by a significant reduction in vessel area in WT mice (3.23 ± 0.25/(100 mm)^2^; *P* < 0.05) at 3 dpi as compared with sham (4.90 ± 0.63/(100 mm)^2^) mice (Fig. 1g). Similar but not significant trends in vessel area reduction were observed in ephrinB3^-/-^ and EphB3^-/-^ mice at 3 dpi as compared to their respective shams.

To provide a second and more sensitive analysis of ECs numbers, we quantified cortical vascular endothelial cells (cvECs) from sham and CCI injured mice using flow cytometry (Fig. 1h). The gating strategy was based on using forward and side scatter to exclude cellular debris and select for homogeneity of size and granularity of individual cells isolated from a cortical hemisphere. The selection marker CD45 was used to exclude infiltrating leukocytes and residential microglia from the analysis, where cvECs were also identified as CD45^-^/CD144^+^ cells^36^. We observed a 36% reduction in the number of cvECs in the WT CCI injured (1.65 × 10^5^ ± 140 cell/μL; *P* < 0.001) cortex at 3 dpi as compared with WT sham (2.59 × 10^5^ ± 230 cell/μL) cortex (Fig. 1i). Conversely, EphB3^-/-^ mice had only a 19% reduction in cvEC numbers after CCI injury (2.27 × 10^5^ ± 280 cell/uL) that was not significantly different from EphB3^-/-^ sham (2.81 × 10^5^ ± 410 cell/μL) mice. In fact, the number of cvECs in CCI injured EphB3^-/-^ mice was significantly (*P* < 0.05) greater then CCI injured WT mice. EphrinB3^-/-^ mice showed a significant 28% reduction in cvECs numbers (*P* < 0.05) similar to WT mice after CCI injury.

### EphB3 signaling in cvECs leads to increased cell death but no effect on proliferation after CCI injury

**Fig. 2:**
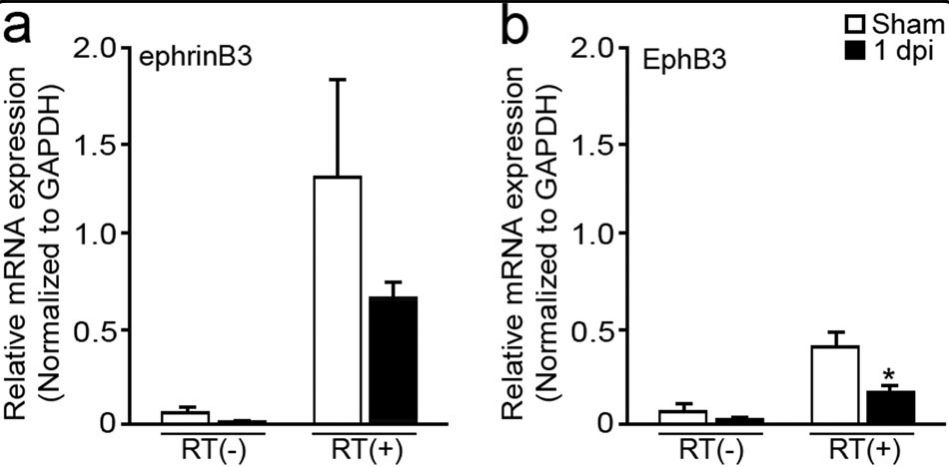
EphB3 and ephrinB3 mRNA are down regulated in the cortex at 1 dpi as compared to sham controls from brain ECs isolated by FACS using quantitative RT-PCR analysis EphrinB3** a** and EphB3 **b** mRNA are downregulated in ECs isolated from the mouse cortex at 1 dpi using FACS and quantitative RT-PCR as compared to sham controls. RT(-) reflects no RT product. *N*-values are as follows: WT sham (*n* = 4); WT CCI (*n* = 3) (run in triplicate). *P < 0.05

EphB3 has been shown to be expressed in various CNS cell types and has both anti-proliferative and pro-apoptotic functions after CCI injury^19,20,37^; however, their potential role in cvECs is unknown. To examine the expression of ephrinB3 and EphB3 in the endothelial population after CCI injury, we isolated cvECs using FACS and harvested mRNA for quantitative (q)RT-PCR analysis at 1 dpi. mRNA levels were measured since commercial antibodies are non-specific and/or of poor quality. Both ephrinB3 and EphB3 mRNA are detected in sham cvECs and show ~50–60% reduction after CCI injury (Fig. 2). This corresponds to reductions in whole cortical protein levels previously observed at 3 dpi^20^.

**Fig. 3:**
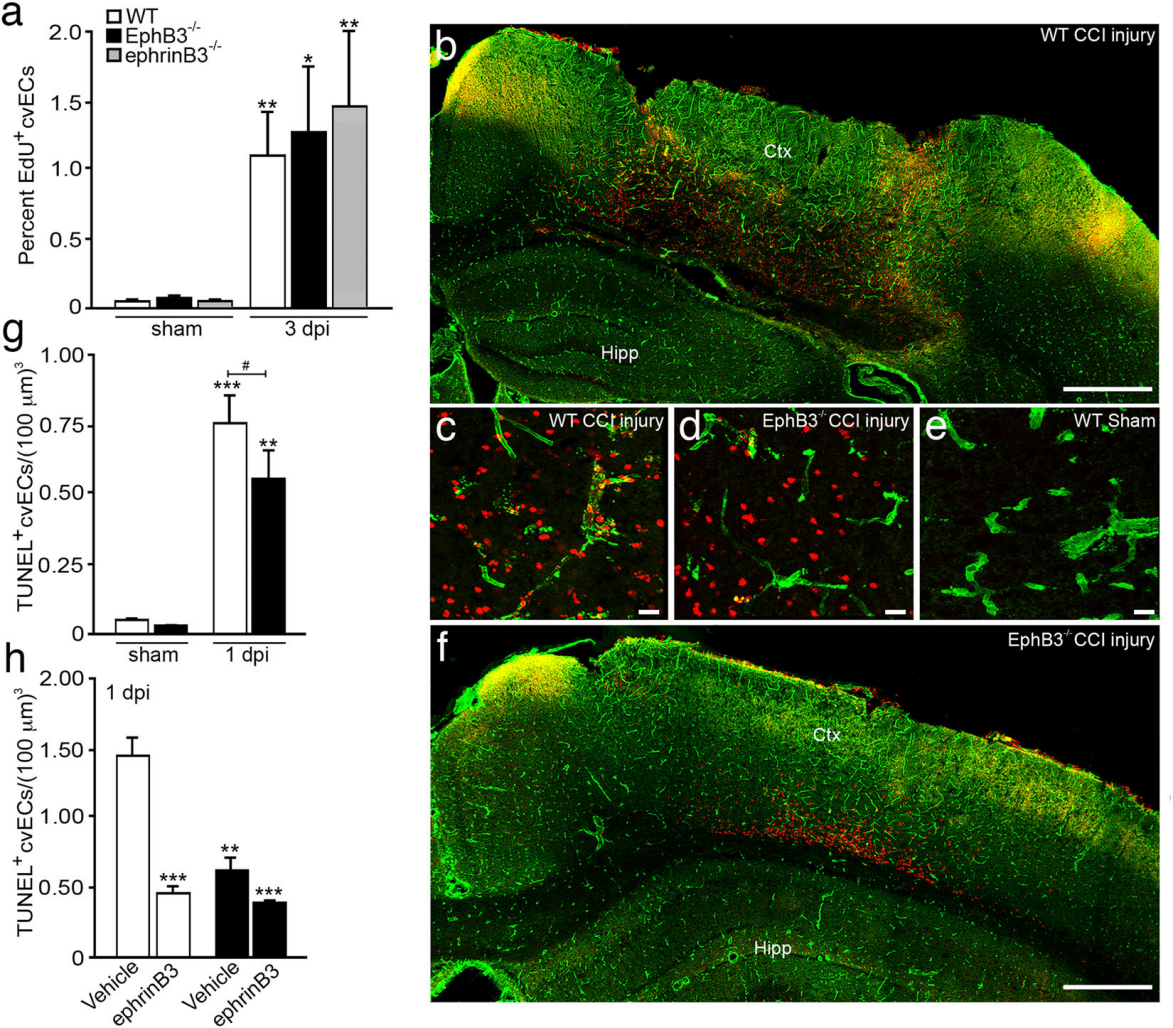
EphB3 regulates cortical vascular endothelial cell (cvEC) death but not proliferation **a** Flow cytometric analysis of EdU^+^ CD45^-^/CD144^+^ cvECs showed increased proliferation at 3 dpi for all genotypes, but no significant difference between genotypes. *N*-values for panel a are as follows: WT sham (*n* = 12); WT CCI (*n* = 15); EphB3^-/-^ sham (*n* = 5); EphB3^-/-^ CCI (*n* = 6); ephrinB3^-/-^ sham (*n* = 14); ephrinB3^-/-^ CCI (*n* = 15). **b** Low-magnification representative image of a TUNEL (red) and Glut-1 (green) co-labeled WT cortex at 1 dpi. High-magnification representative image of TUNEL co-expression with Glut-1-positive cvECs in CCI injured WT **c** and EphB3^-/-^**d** mice as compared to WT sham controls **e**. **g** Quantified TUNEL^+^/Glut-1^+^ cvECs show increased numbers at 1 dpi; however, EphB3^-/-^ cortices are reduced as compared with WT mice. *N*-values for panel **g** are as follows: WT shams (*n* = 3); WT CCI (*n* = 6); EphB3^-/-^ sham (*n* = 3); EphB3^-/-^ CCI (*n* = 6). **h** Administration of recombinant ephrinB3 to the ipsilateral injured cortex for 24 h resulted in a significant reduction in TUNEL labeling in WT but not EphB3^-/-^ mice. *N*-values for panel **h** are as follows: WT CCI-vehicle (*n* = 4); WT CCI-ephrinB3 infusion (*n* = 4); EphB3^-/-^ CCI-vehicle (*n* = 3); EphB3^-/-^ CCI-ephrinB3 infusion (*n* = 4) *^,#^*P* < 0.05; **P < 0.01; ***P < 0.001. *Compared to their respective genotype-specific controls (except in **h**, all compared to WT vehicle-treated group). ^#^Compared to WT CCI injured mice. Bar is 500 μm in **b**, **f** and 20 μm in **c**–**e**

To determine whether the increase in cvEC numbers observed in the CCI injured EphB3^-/-^ mice resulted from increased proliferation, we examined the percent of EdU^+^ cvECs using flow cytometry at 3 dpi. CCI injury led to greater numbers of proliferating cvECs that was similar between all genotypes (Fig. 3a). This suggests that EphB3 does not have anti-proliferative functions in cvECs as shown for neural stem/progenitor cells^19,37,38^. We next examined cvEC death using non-biased stereological measurements of TUNEL^+^/Glut-1^+^ cells in the WT and EphB3^-/-^ mice at 1 dpi. In our current studies we observed a large increase in overall TUNEL labeling (red) in the WT CCI injured cortex (Fig. 3b). High-magnification stereological assessment was used to quantify the number of TUNEL^+^ nuclei that co-labeled with Glut-1-positive cvECs between WT and EphB3^-/-^ mice (Fig. 3c–e). In CCI injured EphB3^-/-^ mice, dramatically less TUNEL-labeling was observed (Fig. 3f), and little to no TUNEL-labeling was observed in sham controls (Fig. 3e). Stereological quantification of specifically cvECs showed a ~1.5-fold increase in TUNEL-positive cvECs after CCI injury; however, the number of TUNEL-positive cvECs was significantly (*P* < 0.05) reduced in EphB3^-/-^ mice (0.56 ± 0.11 cvECs/(100 μm)^3^) at 1 dpi as compared with WT (0.76 ± 0.11 cvECs/(100 μm)^3^) mice (Fig. 3g).

To verify that EphB3 functions as a pro-apoptotic death receptor in the absence of its ligand, we administered recombinant ephrinB3 proteins^26^ or vehicle directly into the site of injury using mini osmotic pumps. We observed a significant ~68% reduction in TUNEL^+^/Glut-1^+^ cvECs in the WT CCI injured mice infused with 80 μg/kg/day ephrinB3 for 24 h (Fig. 3h). In the absence of EphB3 we observed similar reductions in both vehicle and ephrinB3 infused mice, suggesting that the ephrinB3 effects are specifically EphB3-mediated. Altogether, our findings support a pro-apoptotic dependence receptor function for EphB3 in cvEC death after CCI injury, where eliminating EphB3 signaling leads to increased cvEC numbers.

### HUVECs express EphB3 and undergo dependence receptor-mediated cell death following stress

**Fig. 4:**
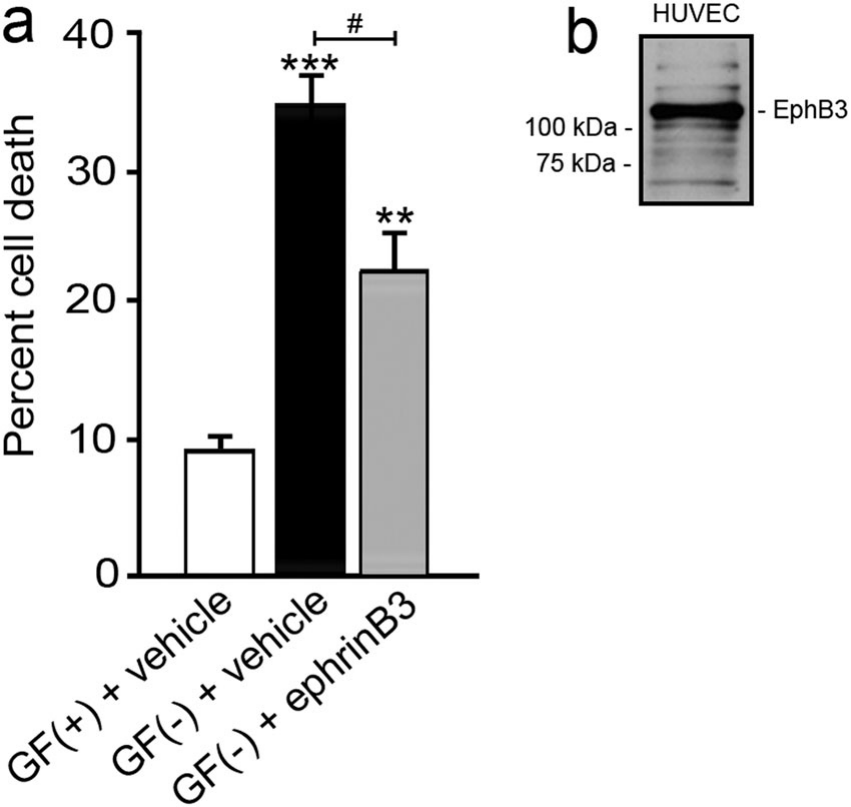
Growth factor (GF) removal increased cell death in EphB3 expressing HUVECs **a** Quantitative analysis of HUVECs grown with GFs (GF( + ) + vehicle) (*n* = 8), without GFs (GF(-) + vehicle) (*n* = 8), and without growth factor in the presence of 1 mg/mL ephrinB3 (GF(-) + ephrinB3) (*n* = 8). **b** Western blot analysis of EphB3 expression in HUVECs using an anti-EphB3 antibody

We initially examined whether dependence receptor functions were observed in primary cvECs; however, EphB3 and ephrinB3 were dramatically downregulated in cultured cvECs (Supplementary Fig. 1). This reduction in EphB3 expression as a result of prolonged culturing, makes it difficult to evaluate dependence receptor functions in primary mouse cvECs. Alternatively, we examined EphB3 functions in cultured HUVECs where detectable levels of EphB3 protein were observed by western blot analysis (Fig. 4b). To examine dependence receptor functions, we induced HUVEC stress by withdrawing growth factor (GF) supplements to enhance Eph-mediated cell death as shown for other cells^20,21,23^. We observed a significant increase in cell death 48 h after GF removal, where 34.9 ± 2.4% of HUVECs were Trypan blue positive (Fig. 4a) as compared to 9.8 ± 1.2% cell death in cultures containing GFs. Administration of ephrinB3 led to a significant improvement in cell survival after GF removal, where 22.5 ± 2.9% cell death was observed. Altogether, our findings provide strong support for the cell autonomous functions of EphB3 in dependence receptor-mediated cell death in ECs.

### Deficiencies in EphB3 and ephrinB3 reduce BBB breakdown after CCI injury

**Fig. 5:**
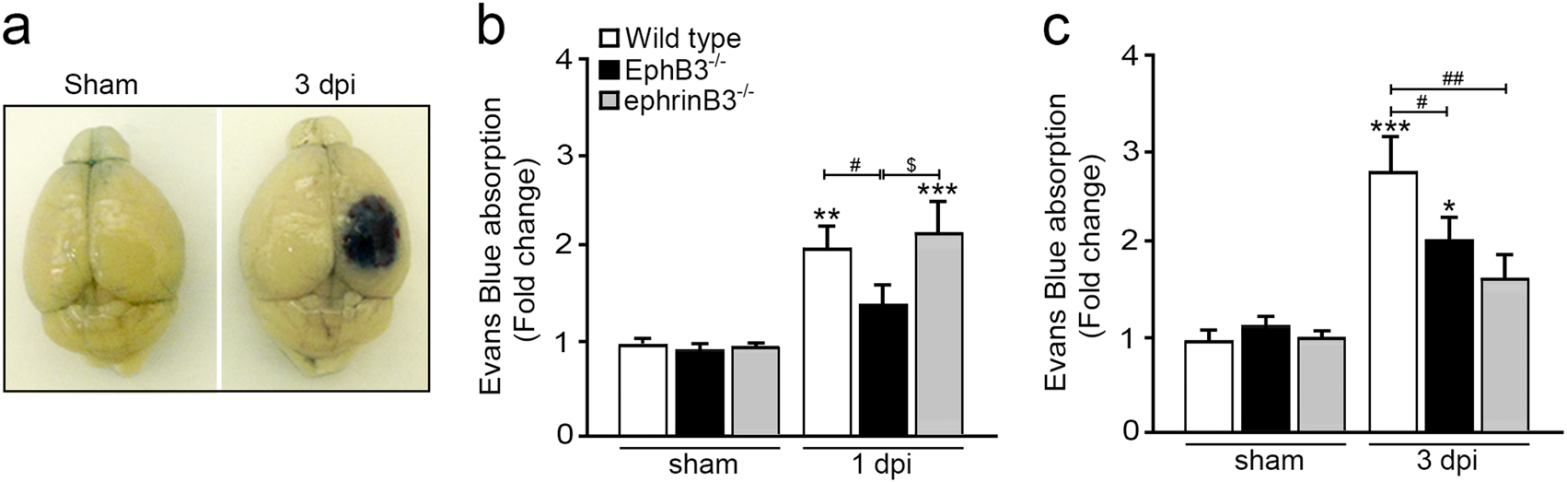
CCI injury led to increased BBB permeability that was reduced in the absence of EphB3 and ephrinB3 **a** Example of Evans blue (EB) extravasation at the injury site of a representative WT mouse brain at 3 dpi as compared with sham control. **b** Quantification of EB absorption showed significant increase in WT and ephrinB3^-/-^ mice that was not observed in EphB3^-/-^ mice at 1 dpi. *N*-values for panel **b** are as follows: WT sham (*n* = 5); WT CCI (*n* = 13); EphB3^-/-^ sham (*n* = 5); EphB3^-/-^ CCI (*n* = 11); ephrinB3^-/-^ sham (*n* = 5); ephrinB3^-/-^ CCI (*n* = 15). **c** Quantification of EB absorption showed significant increase in WT and EphB3^-/-^ mice that was not observed in ephrinB3^-/-^ mice at 3 dpi, where EphB3^-/-^ and ephrinB3^-/-^ mice were significantly reduced as compared with WT CCI injured mice. *N*-values for panel **c** are as follows: WT sham (*n* = 11); WT CCI (*n* = 14); EphB3^-/-^ sham (*n* = 5); EphB3^-/-^ CCI (*n* = 8); ephrinB3^-/-^ sham (*n* = 6); ephrinB3^-/-^ CCI (*n* = 6). *^,#,$^*P* < 0.05; **^,##^*P* < 0.01; ***P < 0.001. *Compared to their respective genotype specific controls. ^#^Compared to WT CCI injured mice. ^$^Compared to EphB3^-/-^ CCI injured mice

To begin examining the role of eprhinB3 and EphB3 in BBB integrity, we first evaluated the BBB permeability to macromolecules using an Evans blue (EB) brain tissue extravasation assay in sham and CCI injured WT mice at 1 and 3 dpi (Fig. 5a). Values were measured as the amount of EB extravasation into the CCI injured cortex and normalized to the non-injured contralateral cortex to account for variability in possible vessel development between groups. No differences were observed in BBB leakiness between WT, EphB3^-/-^, ephrinB3^-/-^ sham mice at 1 or 3 dpi (Fig. 5b, c). At 1 dpi, WT CCI injured mice showed a significant ~ 2-fold increase (*P* < 0.01) in EB extravasation as compared with WT sham mice (Fig. 5b). Similar increases were observed in CCI injured ephrinB3^-/-^ mice at 1 dpi; however, EphB3^-/-^ mice showed a significant reduction (*P* < 0.05) in EB extravasation from both WT and ephrinB3^-/-^ CCI injured mice. At 3 dpi, CCI injured EphB3^-/-^ and ephrinB3^-/-^ mice were both significantly reduced (*P* < 0.05 and *P* < 0.01, respectively) from CCI injured WT mice (Fig. 5c). The differential response between ephrinB3^-/-^ and EphB3^-/-^ mice at 1 and 3 dpi may suggest that the mechanism of action may involve more than a direct ligand–receptor interaction. In fact, the ephrin/Eph family are known to be promiscuous binding partners where ephrinB3 can interact with other Ephs (such as EphA4) and EphB3 can interact with other B-class ephrins^26,39^. Altogether, these findings suggest that both EphB3 and eprhinB3 are involved in BBB permeability after TBI.

**Fig. 6:**
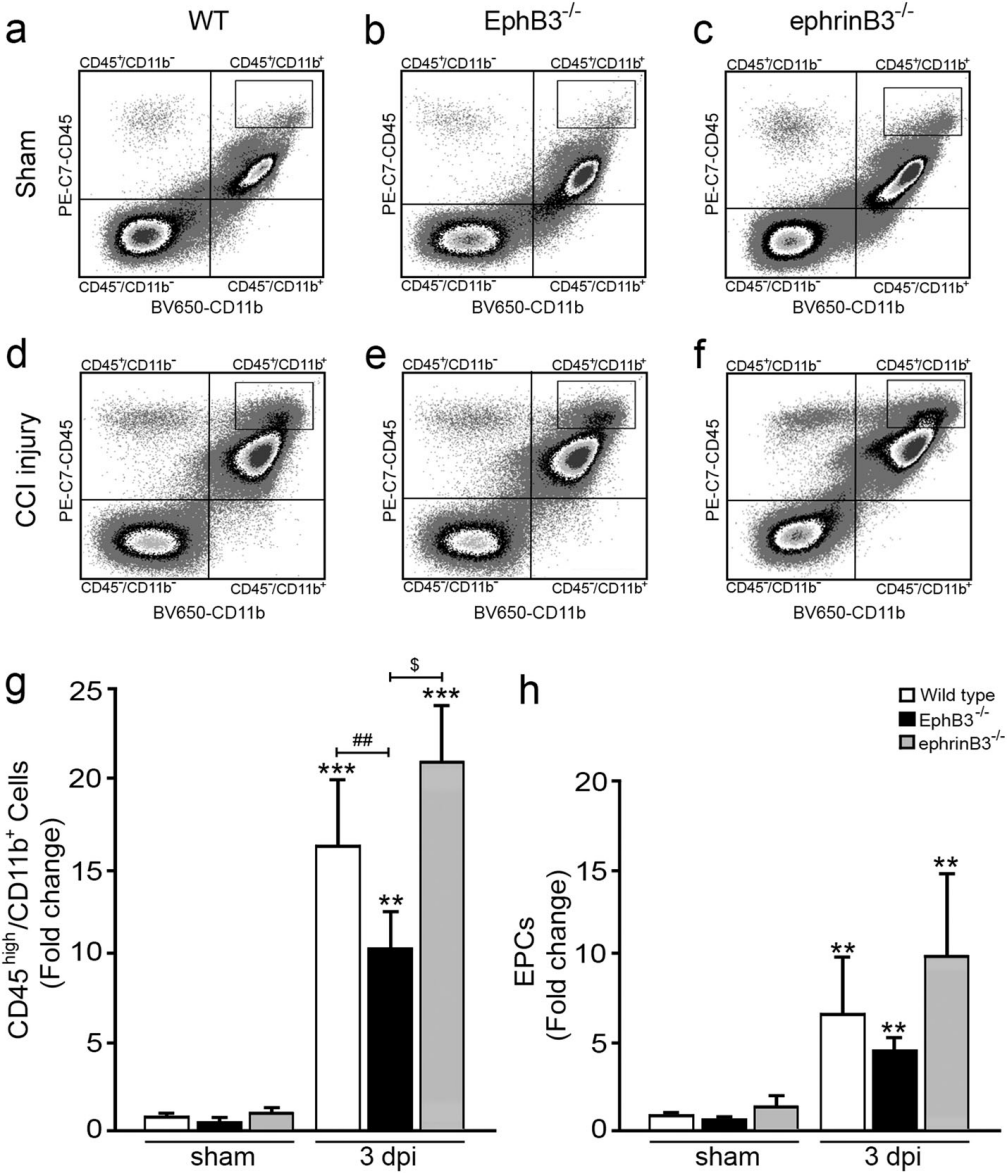
Macrophage and endothelial progenitor cell (EPC) infiltration was reduced in the absence of EphB3 **a**–**f** Flow cytrometry analysis of CD45 and CD11b expressing cells in WT, EphB3^-/-^, ephrinB3^-/-^ mouse brains at 3 dpi as compared with sham controls, where CD45^high^/CD11b^+^ cells represent infiltrating peripheral macrophages. **g** Quantification of CD45^high^/CD11b^+^ peripheral macrophages showed increased levels after CCI injury that are significantly reduced in EphB3^-/-^ mice as compared with WT and ephrinB3^-/-^ mice. *N*-values for panel **g** are as follows: WT sham (*n* = 12); WT CCI (*n* = 14); EphB3^-/-^ sham (*n* = 8); EphB3^-/-^ CCI (*n* = 9); ephrinB3^-/-^ sham (*n* = 12); ephrinB3^-/-^ CCI (*n* = 12). **h** Quantification of CD45^-^/CD144^-^/CD309^+^/CD133^+^ EPCs showed increased levels after CCI injury and a trend towards reduced levels in EphB3^-/-^ mice. *N*-values for panel **h** are as follows: WT sham (*n* = 12); WT CCI (*n* = 15); EphB3^-/-^ sham (*n* = 5); EphB3^-/-^ CCI (*n* = 6); ephrinB3^-/-^ sham (*n* = 14); ephrinB3^-/-^ CCI (*n* = 15). ^$^*P* < 0.05; **^,##^*P* < 0.01; ***P < 0.001. *Compared to their respective genotype specific controls. ^#^Compared to WT CCI injured mice. ^$^Compared to EphB3^-/-^ CCI injured mice

We next examined whether deficiencies in EphB3 or ephrinB3 affected BBB permeability to infiltrating peripheral cells^36^. Infiltrating macrophages express high levels of CD45 (i.e., CD45^high^) and CD11b markers, which were significantly increased in all groups at 3 dpi (Fig. 6a–g). Comparison of CCI injured mice showed a significant reduction in macrophage infiltration in EphB3^-/-^ mice as compared to WT (*P* < 0.01) and ephrinB3^-/-^ (*P* < 0.05) mice (Fig. 6g), which is similar to that observed in the EB extravasation assay at 1 dpi. We also examined infiltration of CD45^-^/CD144^−^/CD309^+^/CD133^+^ EPCs, which are known to play a role in the repair of damaged vessels^40–42^. Similar to infiltrating macrophages, there is a dramatic increase in the number of infiltrating EPCs at 3 dpi as compared with sham controls, where a trend towards fewer EPCs were observed in the injured cortex of EphB3^-/-^ mice (Fig. 5h). We also determined that reduced cell numbers observed in EphB3^-/-^ CCI injured mice did not result from reduced proliferative responses of CD45^high^/CD11b^+^ or EPCs, since WT, ephrinB3^-/-^ and EphB3^-/-^ mice had equal enhancement in the number of proliferating cells after CCI injury (Supplementary Fig. 2). Altogether, these results demonstrate that EphB3 signaling plays a significant role in BBB breakdown after CCI injury, which may partially involve interactions with ephrinB3 and/or other ephrin family members.

### Disruption of EphB3 leads to alterations in the gliovascular unit

**Fig. 7:**
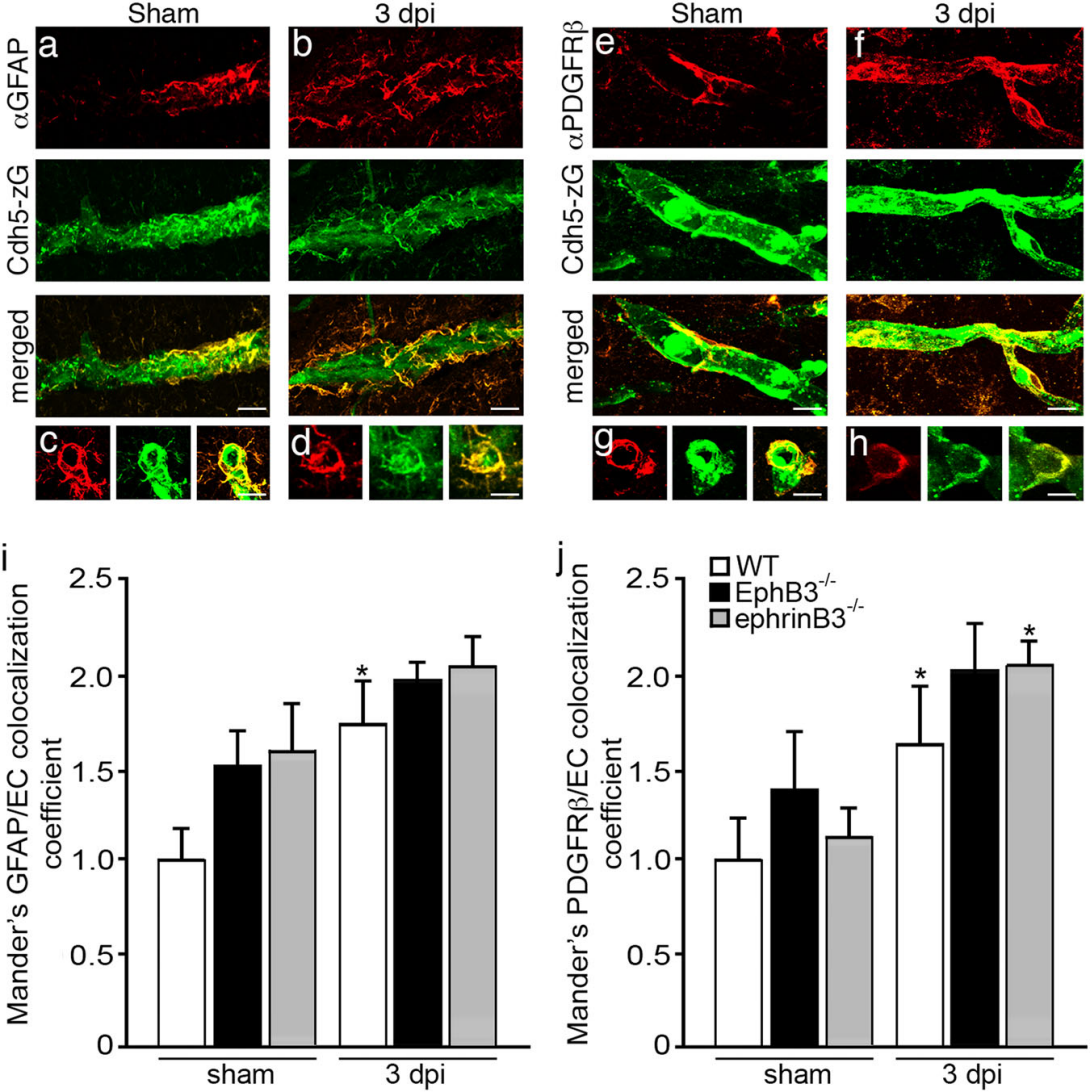
CCI injury leads to increase glial membrane interactions with cvECs **a**–**d** Confocal analysis of GFAP-labeling (astrocytes, red) in Cdh5-zG (green) expressing cvECs in CCI injured mice show increased co-labeling in vessel segment **b** and cross-section **d** at 3 dpi as compared to WT sham controls **a**, **c**. **e**–**h** Confocal analysis of PDGFRβ-labeling (pericytes, red) in Cdh5-zG expressing cvECs in WT sham and CCI injured mice show co-labeling in vessel segment **e**, **f** and cross-section **g**, **h** at 3 dpi as compared to sham controls. **i** Mander’s coefficient measures the level of colocalization between fluorophores, where increased trends of astrocyte-cvEC membrane interactions were observed in sham EphB3^-/-^ and ephrinB3^-/-^ mice as compared with WT mice, but also in CCI injured mice. No difference was observed in CCI injured vs. sham EphB3^-/-^ or ephrinB3^-/-^ mice. *N*-values for panel **i** are as follows: WT sham (*n* = 8); WT CCI (*n* = 10); EphB3^-/-^ sham (*n* = 8); EphB3^-/-^ CCI (*n* = 10); ephrinB3^-/-^ sham (*n* = 7); ephrinB3^-/-^ CCI (*n* = 9). **j** Increased pericyte-cvEC membrane interactions were not observed between sham mice, but WT, EphB3^-/-^, and ephrinB3^-/-^ CCI injured mice were increased as compared to their respective sham controls. *N*-values for panel **j** are as follows: WT sham (*n* = 8); WT CCI (*n* = 11); EphB3^-/-^ sham (*n* = 8); EphB3^-/-^ CCI (*n* = 10); ephrinB3^-/-^ sham (*n* = 6); ephrinB3^-/-^ CCI (*n* = 7). *P < 0.05 as compared to their respective genotype specific controls. Bar is 10 μm in **a–h**

The gliovascular unit is a functionally interacting group of cells that are represented by astrocytes and pericytes that ensheath brain endothelium^43^. This glial-EC membrane association plays important roles in both brain homeostasis and vascular repair. To examine membrane interactions between cvECs and either astrocytes or pericytes, we immunostained Cdh5-zG mice with either anti-GFAP or anti-PDGFRβ antibodies, respectively, and measured the level of membrane interactions using z-stack confocal imaging and FIJI-imageJ analysis (Fig. 7). The Mander’s split coefficient determines the proportion of colocalization between two fluorescent channels. Compressed z-stack images of vessels in the peri-lesional cortex showed interactions of vessels (green) with astrocytes (red) in the sham and 3 dpi animals (Fig. 7a–d) as well as interactions with pericytes (red) (Fig. 7e–h). In sham mice, we observed no significant difference in the amount of astrocytic or pericytic membranes that interact with cvECs in WT, EphB3^-/-^ and ephrinB3^-/-^ mice (Fig. 7i–j), although there were large trends in the absence of EphB3 and ephrinB3. After CCI injury, astrocyte-cvEC interactions where significantly (*P* < 0.05) increased ~ 1.75-fold in WT mice, whereas EphB3^-/-^ and ephrinB3^-/-^ mice showed similar trends that were not significantly increased from their respective sham controls (Fig. 6i). Analysis of pericyte membranes using anti-PDGFRβ showed similar increased pericyte-cvEC association after CCI injury in all three genotypes (Fig. 7j). These observations suggest that CCI injury leads to enhanced glial membrane interactions with damaged vessels, which may represent a reparative response to TBI.

## Discussion

TBI is a dynamic and progressive disorder leading to vascular disruption that underlies many of the functional deficits observed in TBI patients. Vessel damage and/or disruption of the BBB are acute responses to the initial trauma and are prevalent in most brain injuries. Cortical vessels are composed of cortical vascular endothelial cells (cvECs) that form tight junctions and associate with glial end-feet and neuronal projections to form gliovascular and neurovascular units, which together participate in establishing an intact BBB. In this study, we examine signals that mediate BBB stability, vascular repair and re-growth where EphB3 receptors play a deleterious role in recovery. In EphB3-deficient mice (EphB3^-/-^ mice) there are greater numbers of surviving cvECs that result in increased vessel sparing following CCI injury, which reflects a novel dependence receptor mechanism of cell death for EphB3 in cvECs. This is supported by our observations that ephrinB3 administration increases cvEC and HUVEC survival, where by definition ligand-receptor interactions block dependence receptor-mediated cell death. We also found reduced BBB permeability to macromolecules and infiltrating peripheral cells in the absence of EphB3. Additionally, the integrity of the BBB involves membrane interactions between brain ECs and glia, where the absence of EphB3 or ephrinB3 led to increased association of astrocytic end-feet with cvECs in non-pathological conditions. After CCI injury, a significant enhancement in astrocyte and pericyte membrane association with cvECs occurs in WT mice, which is not seen in EphB3^-/-^ or ephrinB3^-/-^ mice. Altogether, our findings support a deleterious role for ephrinB3 and EphB3 in blood vessel integrity after TBI.

In the adult CNS, ECs are thought to be relatively quiescent; however, during adult angiogenesis latent proliferative and apoptotic processes can be re-initiated. In pathological conditions, such as stroke and traumatic injury, the proliferative angiogenic response is activated within hours to days after the initial insult^44,45^, where EC proliferation and EPC infiltration are thought to contribute to vessel repair and regeneration^36,45–50^. Our findings support a small yet significant proliferative response in cvECs within the first 3 days after CCI injury as well as enhanced EPC infiltration at the same time point. We did not observe injury-induced differences in cvEC proliferation or EPC infiltration in the absence of EphB3 or ephrinB3, suggesting that EphB3 signaling is not required for these pro-angiogenic responses. This differs from the anti-proliferative functions for EphB3 in neural progenitor cells after CCI injury, where EphB3 signaling suppresses neural progenitor cell expansion through a p53-mediated pathway^19,38^. Conversely, EphB3 does regulate cvEC survival after CCI injury.

In 2010, EphB3 was first described as a dependence receptor in adult neural progenitor cells, where administration of ephrinB3 or deletion of EphB3 could block cell death in the traumatically injured brain^19^. Since this discovery, neurons^20^ and oligodendrocytes^21^ have also been shown to undergo EphB3-mediated cell death after CNS injury. Here, we describe a dependence receptor role for EphB3 in cvECs where pro-apoptotic mechanisms regulate vascular integrity after CCI injury. In the absence of EphB3, greater numbers of surviving cvECs were observed at 3 dpi and fewer TUNEL-positive ECs were observed at 1 dpi, supporting the role of EphB3 in regulating EC survival after CCI injury. A characteristic that is unique to dependence receptors, as compared to other death receptors, is that ligand activation blocks receptor-mediated cell death. In the CCI injured brain, acute cellular disruption may be the first event underlying dependence receptor mechanisms of cell death, considering EphB3 receptor expression is not reduce until at least 24 hpi, but acute necrosis leads to reduced cell–cell interactions. Since ephrin ligands and Eph receptors are both membrane-bound, this early cell death would result in non-ligated receptors and, in turn, an environment that propagates dependence receptor cell death mechanisms. Infusion of soluble ephrinB3 can reverse cell death in wild-type mice but not in EphB3^-/-^ mice, supporting the dependence receptor functions of EphB3–ephrinB3 interactions. This protective response was also observed in stressed HUVECs cultured in the presence of ephrinB3. Our findings suggest that acute damage to blood vessels likely involves pro-apoptotic mechanisms as a result of the activation of dependence receptor signals.

The BBB also participates in regulating vessel stability after CNS injury, where gliovascular and neurovascular units contribute to the formation of this multicellular structure. The gliovascular unit involves a direct association of ECs with pericytes and astrocytes^43,51^. In addition to ECs, ephrins and Ephs are also expressed by both astrocytes and pericytes, suggesting that the cells that make up the gliovascular unit may communicate through bidirectional signaling mechanisms known to occur between ephrins and Eph receptors^14,52,53^. It’s less clear whether ephrins and Ephs are contained in astrocytic- or pericytic-end-feet, although they have been localized to the glial filopodia and axonal sprout and regulate cytoskeletal stability^54,55^. Our findings showing increased glial-EC membrane association occurring in the absence of EphB3 or ephrinB3 supports a role for EphB3 signaling in regulating astrocytic end-feet ensheathing. Reduced ephrinB3 and EphB3 EC expression after CCI injury also supports the observed enhanced astrocyte-EC membrane interactions. One possibility is that the brain’s response to traumatic injury is to enhance glial ensheathing to minimize BBB damage, where changes in ephrinB3/EphB3 signaling may contribute to this response. Interestingly, we observe a differential response in BBB integrity in the absence of ephrinB3 at 1 and 3 dpi but not in the absence of EphB3. The likely probability for these differences would result from the possibility that EphB3 interacts with ephrinB1 and/or ephrinB2. In fact, ephrinB1 levels have been shown to be upregulated in whole brain extracts at 3 dpi^56^ when ephrinB3 levels are reduced^20^. Agrin proteoglycan are also known to regulate astrocyte end-feet formation with ECs and accumulates in brain microvessels at the time of BBB tightening^43,57,58^. In the traumatic injured brain, agrin expression was increased during the first week following a fluid percussion injury in rats^59^. In a separate study, agrin was found to induce EphB1 receptor clustering and activation in erythroblasts, which led to activation of α5β1 integrins and enhanced cell–cell adhesion^60^. We observed enhanced astrocyte end-feet wrapping of cvECs at a similar time period; however, additional studies are needed to determine whether this is an agrin/EphB3-mediated event. Overall EphB3 signaling is an important negative regulator of BBB integrity after acute traumatic CNS injury, where blocking these signals could lead to improved recovery.

Vessel repair is known to occur in the injured adult CNS, yet intrinsic vessel regeneration is most often insufficient for functional recovery. One possible contributing factor is that the angiogenic response may be slanted towards an anti-regenerative state. Pro-angiogenic factors, such as vascular endothelial growth factor (VEGF) are known to stimulate EC proliferation, migration, and vascular permeability^61^. This led to VEGF administration studies in TBI, where it’s been shown to enhance both angiogenesis and neurogenesis^62,63^. However, VEGF has also been shown to have deleterious effects on pericyte function and vessel maturation, where VEGF administration reduced pericyte coverage of nascent vascular sprouts that led to vessel destabilization^64^. Anti-regenerative factors also contribute to the angiogenic potential in the injured CNS, including families of pro-apoptotic factors, such as TNFα and Fas receptor^65^. It is known that apoptosis in the developing vascular system plays an important role in tissue remodeling^66^. In particular, blood vessel morphogenesis requires vessel growth and regression to properly form the vascular network in the developing nervous system, during wound healing and tumorgenesis^11,67,68^. In the absence of TNFα and Fas, CCI injured mice have decreased lesion size that correlated to improvements in motor and spatial memory functions^65^. A secondary role for TNFα is also known for its pro-inflammatory roles that can lead to cytokine-mediated BBB breakdown and subsequent CNS tissue damage^69^. Our studies suggest that EphB3-mediated cvEC death represents a new class of pro-apoptotic factors that also participate in limiting angiogenesis in the traumatically injured brain.

## Electronic supplementary material

Supplementary Figure 1

Supplementary Figure Legends

Supplementary Figure Legends
