# Supplementary figures and images for: EphB3 signaling induces cortical endothelial cell death and disrupts the blood–brain barrier after traumatic brain injury
Source: Cell Death Dis. 2018 Jan 8;9(1):7. doi: 10.1038/s41419-017-0016-5 (PMC5849033; doi:10.1038/s41419-017-0016-5)

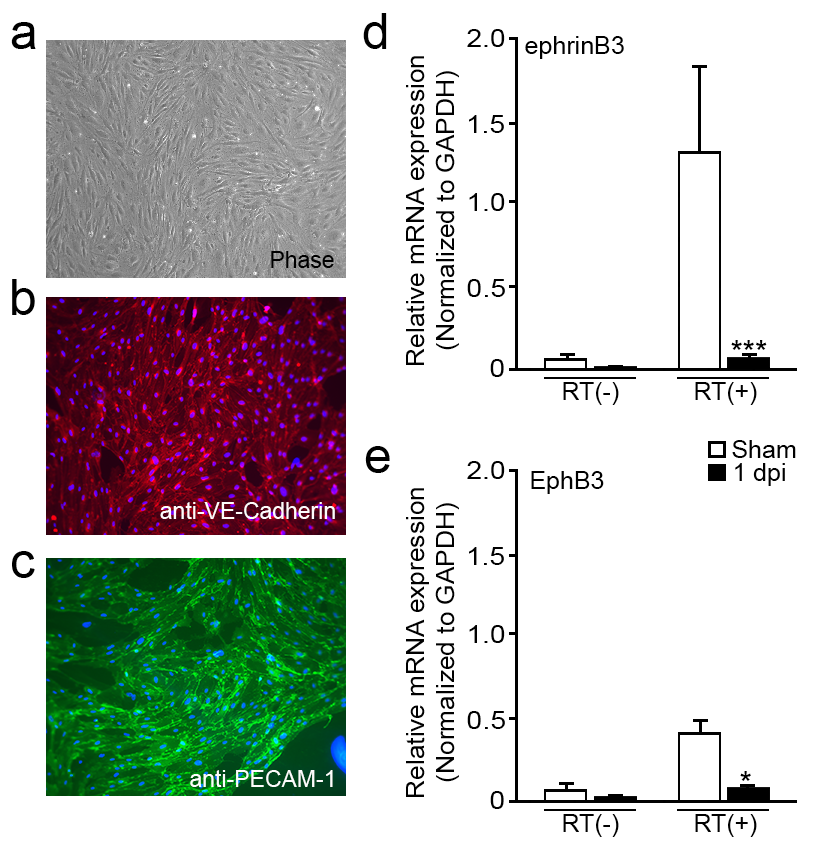

Supplement: Supplementary file 1 — Supplementary Figure 1 [file 41419_2017_16_MOESM1_ESM.tif]

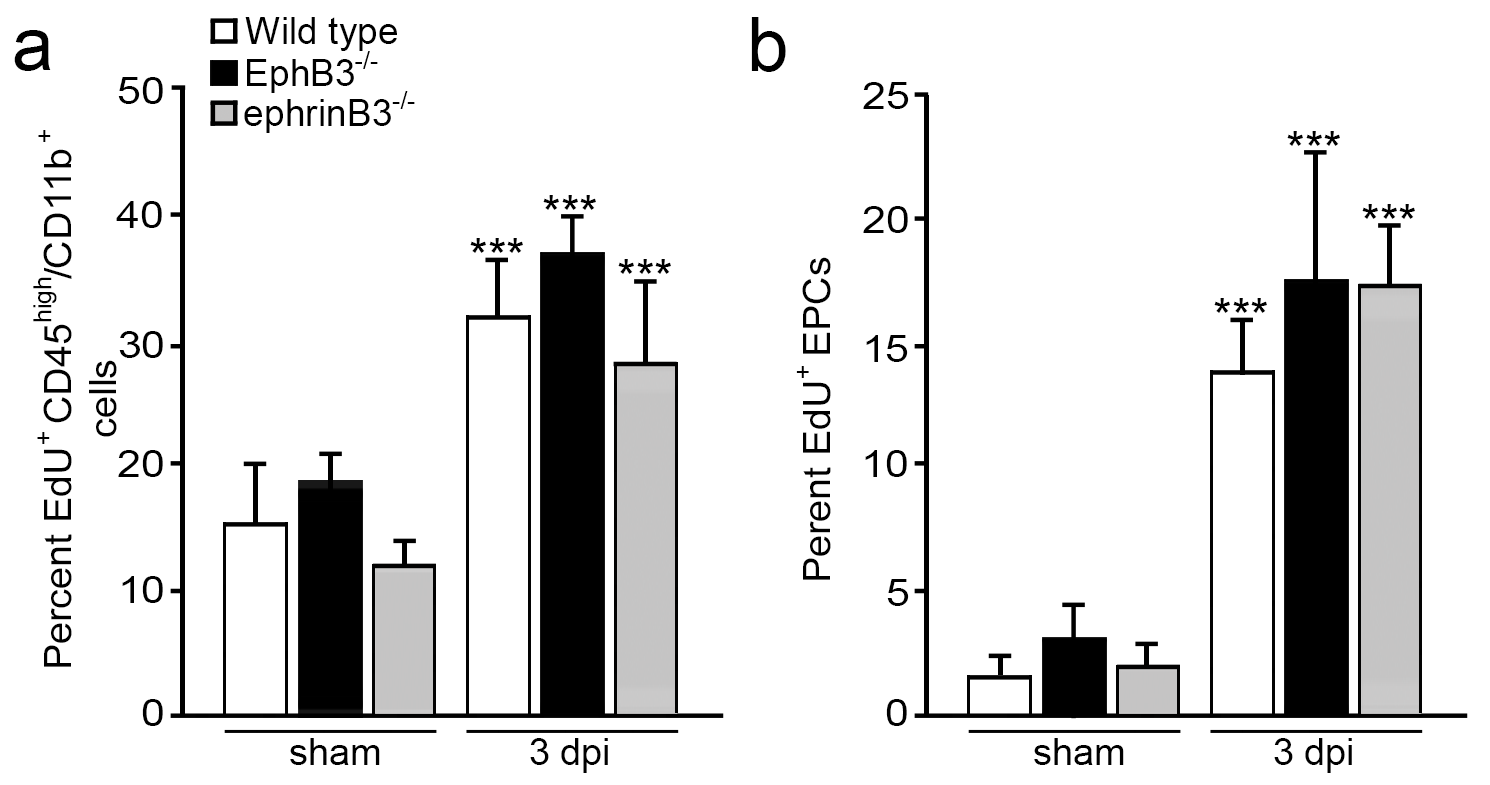

Supplement: Supplementary file 2 — Supplementary Figure Legends [file 41419_2017_16_MOESM2_ESM.tif]
