# Supplementary material for: EphB3 signaling induces cortical endothelial cell death and disrupts the blood–brain barrier after traumatic brain injury
Source: Cell Death Dis. 2018 Jan 8;9(1):7. doi: 10.1038/s41419-017-0016-5 (PMC5849033; doi:10.1038/s41419-017-0016-5)
Supplement: Supplementary file 3 — Supplementary Figure Legends [file 41419_2017_16_MOESM3_ESM.docx]

**Supplementary Figure Legends**

**SFigure 1**. Primary ECs were isolated from the adult murine cortex, purified, and grown for 2-3 weeks to 90% confluency as shown by phase microscopy (a). Brain ECs immunostained with anti-VE-Cadherin (b) or anti-PECAM-1 (c) antibodies shows >95% purity. Quantitative RT-PCR analysis showed significant reductions in ephrinB3 (d) and EphB3 (e) expression as compared to primary non-cultured ECs using FACS isolation. RT(-) reflects no RT product. N-values for panels d and e are as follows: WT sham (n=4); WT CCI (n=3) (run in triplicate). * p<0.05; ***p<0.001.

**SFigure 2**. Macrophage and endothelial progenitor cell (EPC) proliferation was increased in these infiltrating cells after CCI injury. (a) Quantification of EdU^+^ CD45^high^/CD11b^+^ peripheral macrophages showed increased proliferation levels after CCI injury that were not significantly different between genotypes. (b) Quantification of EdU^+^ CD45^-^/CD144^-^/CD309^+^/CD133^+^ EPCs showed increased proliferation levels after CCI injury that were not significantly different between genotypes. N-values for panels a and b are as follows: WT sham (n=12); WT CCI (n=15); EphB3^-/-^ sham (n=5); EphB3^-/-^ CCI (n=6); ephrinB3^-/-^ sham (n=14); ephrinB3^-/-^ CCI (n=15). *** p<0.001 as compared to their respective genotype specific controls.
